# Exploring the impact of southern ocean sea ice on the Indian Ocean swells

**DOI:** 10.1038/s41598-022-16634-0

**Published:** 2022-07-20

**Authors:** Meenakshi Sreejith, Remya P. G., B. Praveen Kumar, Abhijith Raj, T. M. Balakrishnan Nair

**Affiliations:** 1 Ministry of Earth Sciences (MoES), Indian National Centre for Ocean Information Services (INCOIS), Hyderabad, Telangana 500 090 India; 2 School of Ocean Science and Technology, Kerala University of Fisheries and Ocean Studies, Panangad, Cochin, 682506 India

**Keywords:** Ocean sciences, Physical oceanography

## Abstract

The present study analyzes the impact of the Southern Ocean (SO) sea ice concentration on the north Indian Ocean (NIO) wave fields through swells using 6 years (2016–2021) of WAVEWATCH III (WWIII) simulations. We did two experimental runs of WWIII, one with sea ice concentration and winds as the forcing (W3_*with_ice*_) and the second run with only wind forcing (W3_*no_ice*_). Analysis shows the impact of the SO sea ice concentration on northward swell peaks in September–November, coinciding with the maximum sea ice extent in the Antarctic region of the Indian Ocean. W3_*no_ice*_ simulations are biased more by ~ 60% and ~ 37% in significant wave height and period, respectively, against W3_*with_ice*_ when compared with NIO mooring data. W3_*no_ice*_ simulates low-frequency swells and travels fast towards NIO, with implications for operational oceanography. We have shown that the forecasts of the timing of high swell events along NIO coasts can be erroneous by ~ 12 h if the SO sea ice concentration is not included in the model. Further, W3_*no_ice*_ could potentially produce false swell alerts along southeastern Australian coasts. In summary, our study highlights the importance of the SO sea ice concentration inclusion in the wave models to accurately simulate NIO waves.

## Introduction

The sea ice coverage of the Southern Ocean (SO) undergoes a complete seasonal cycle, with minimum ice cover during February and maximum during September–October. The Marginal Ice Zones (MIZ), the transitional zone between the open sea and dense, thick ice packs further south, is an important region where intense coupling between waves, sea ice concentration, ocean, and atmosphere occurs. MIZ is usually not a sharply demarcated edge but a belt comprising ice floes with patches of open water in between them and plays a vital role in regulating global weather and climate patterns. The sea ice in the MIZ regulates the global ocean's carbon and heat uptake^1–3^. They also serve as a crucial connection between the Antarctic regions and the tropical climate and provide a buffer protecting the Antarctic coastline from the SO's intense storms and ocean swells^4,5^.

The variations of sea ice concentration in the Antarctic MIZ impart local and remote changes. Locally generated wind waves travel through the loosely packed sea ice concentration of MIZ and further contribute to more ice breakage^6^, while the attenuation and scattering of waves under the ice ensure its final decay^7,8^. Efforts to integrate the wave-sea ice interactions into the ice/ocean model started long back^9^, but the non-availability of in situ observations for validating the parameterizations hinders success. Major approaches to modelling the sea ice concentration of MIZ vary from simple models with dependence on floe size to direct numerical simulations (DNS) using granular models with either a single floe diameter^10^ or flow diameters from a sample^11^. These studies (and several references therein) underscored the importance of two-way wave-ice interaction, ways of parameterizing it in sea ice models, and its effects. While the role of sea ice concentration in MIZ in regulating the Antarctic polar climate received much scientific attention, its role in modulating wave climate of remote places, like the north Indian Ocean (NIO), through swell generation did not receive the required focus.

Northward of the MIZ, strong year-round westerlies blow over practically an unlimited fetch, generating some of the fiercest waves on the planet^12,13^. These waves travel outside this wave-generating area and northward as long-period swells, modifying the wave climate of the northern hemisphere. Studies in the Indian Ocean (IO) show that the region bounded by 30–50° E and 70–50° S is a strong swell-generating area^14^. Several studies use modelling tools and satellite observations to focus on the IO's swell generation mechanism, propagation route, and energy spread. The swell wave power density of the IO increases from west to east and tends to pile up along the west coast of Australia and then spreads northward of IO^15^. Another modelling study^14^ found that the swell wave height contours in the IO have a pronounced northward feature, and the dominant swell direction is from the south year-round. Generally, there is a convergence of opinion that the SO swells play an important role in determining the wave characteristics of NIO^16^. But, to date, there has not been any study determining the role of sea ice concentration of the SO MIZ in regulating the swell generation and, subsequently, the wave fields of NIO.

The regions in and around the IO are home to roughly 2.6 billion people, which is 40% of the global population, out of which one-third are located within 20 km of the coastline^17^. Hence any modifications to wave climate have an important societal impact on their livelihood. For example, the long period swells from the SO cause flash flooding along the Indian coastline, called the Kallakkadal events^18^, creating much distress in the coastal community. The SO swells cause coastal inundations, hindrance to maritime operations, and possible damage to agriculture by contaminating freshwater reservoirs along coasts. Therefore a thorough knowledge of the modulation of NIO swell characteristics by the SO sea ice cover is significant, especially in the wave projection and forecasting context.

This article is an effort in that direction to estimate and discuss the impact of the SO sea ice concentration on the NIO swell wave characteristics using WAVEWATCH III (WWIII) modelling tools. The rest of the article is organized as follows. Data description and the methodology are described in section two. The differences between the two WWIII simulations we used in this study are discussed in section three and four. We end this article with a summary and discussion in section five.

## Data and methodology

The numerical wave model used in the present study is the WAVEWATCH III- version 6.07 (WWIII) [The WAVEWATCH III Development Group (WWIIIDG), 2016]. INCOIS-WWIII setup has four mosaic grids in nested pattern (Global [0°–360°, 80° S–80° N], Indian Ocean [30° E–120° E, 60° S–30° N], NIO [45° E–100° E, 0°–29° N] and Coastal Ocean [68.5° E–90° E, 5° N–25° N]) with spatial resolution varying from coarser 0.5° global to the finer 0.04° coastal grids. ETOPO1 bathymetry (1′ arc length global relief bathymetry dataset) from National Geophysical Data Centre and global shoreline database (GSHHS- Global Self-Consistent Hierarchical High-resolution Shoreline) is used with an automated grid generation package V2.2^19^ based on an algorithm designed to meld the high-resolution bathymetry with the shoreline database to develop the optimum grid. WWIII integrates the spectral wave energy balance equations in space and time with discretized wave numbers and directions. The present study uses a spectral resolution of 29 frequencies ranging from 0.035 to 0.5 Hz at an increment of 10% in 36 directions. The WWIII package includes many dissipation/input parameterizations, of which switch ST4^20^ is used in this study. As we plan to quantify the impact of the SO sea ice concentration on the swells in NIO, we used the wave-ice parameterization scheme^21^ which provides simple energy flux blocking depending on the local ice concentration.

The wind data used is the 3-hourly European Centre for Medium-range Weather Forecast (ECMWF) winds with a spatial resolution of 0.25° × 0.25°. The daily sea-ice concentration data product from National Snow and Ice Data Centre's Soil Moisture Active Passive (SMAP) L1-L3 Ancillary NOAA Data, Version 1, for 6 years (2016–2021) is used. This dataset, having global coverage and spatial resolution of 0.03° (4 km polar gridded), is used as an input field in WWIII for analyzing the impact of sea ice concentration in the SO on NIO swells.

**Figure 1:**
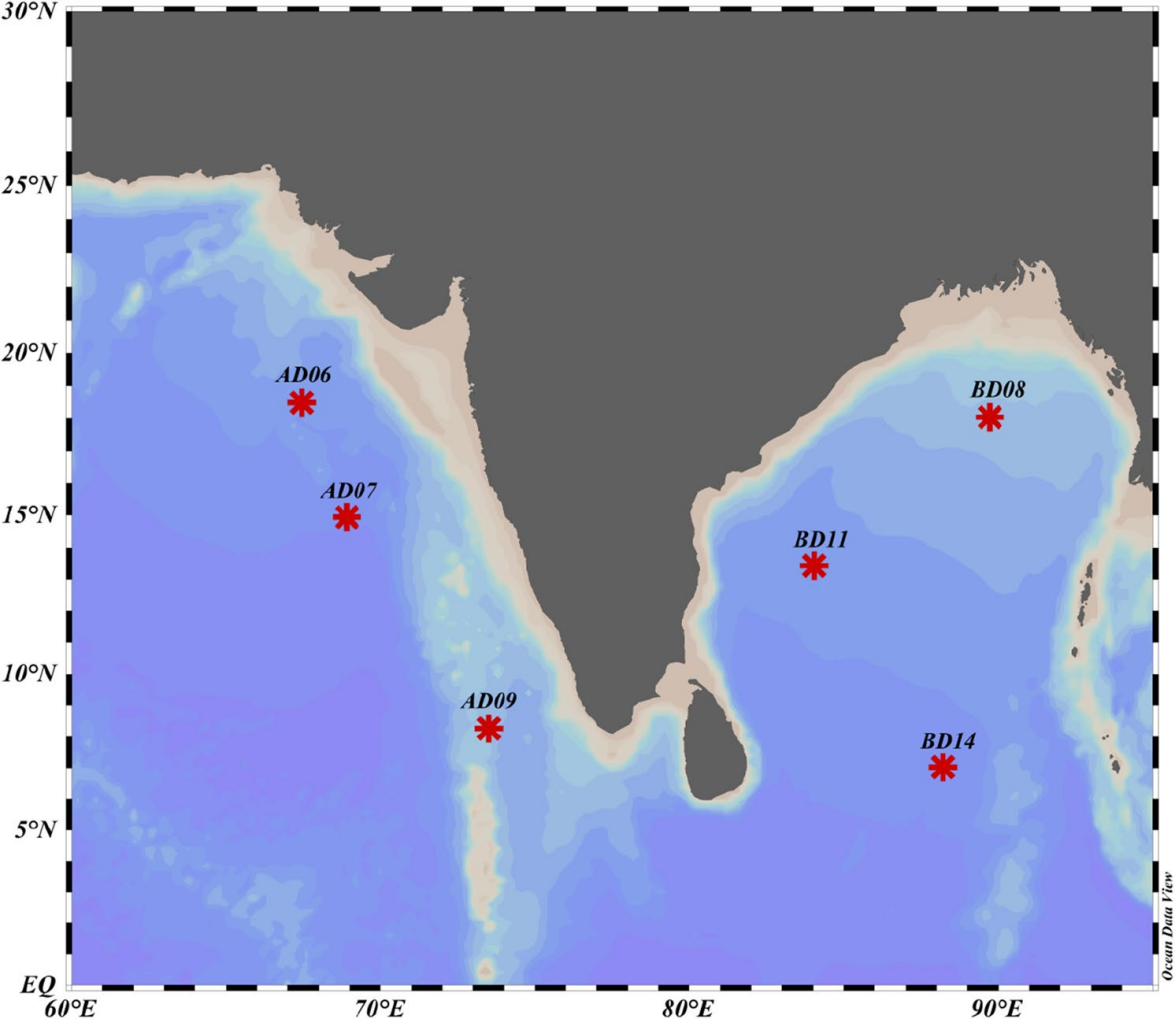
Map showing locations of deep water moored buoy locations in the North Indian Ocean (NIO).

The model results are then validated against data from six offshore moored buoys from NIO (Fig. 1), deployed by the National Institute of Ocean Technology (NIOT, Chennai, India)^22^ under the National Data Buoy Program (NDBP). These moorings are named as AD06 (67.45° E, 18.5° N), AD07 (68.9° E, 15° N), AD09 (73.366° E, 8.25° N), all located in the Arabian Sea; BD08 (89.67° E, 18.17° N), BD11 (83.96° E, 13.48° N), and BD14 (88.08° E, 6.99° N) all in Bay of Bengal basin. Statistical comparison of model results with observation data is made using Pearson's linear correlation coefficient (r), Bias, Root Mean Square Error (RMSE), and Scatter Index (SI). Altimeter data from Jason-2 has been used for the validation of Hs in the SO. Jason-2 covers a swath width of 6–7 km with repeat cycles at about 10 days.

## Difference between with and without SO sea ice concentration in wave simulations

**Figure 2:**
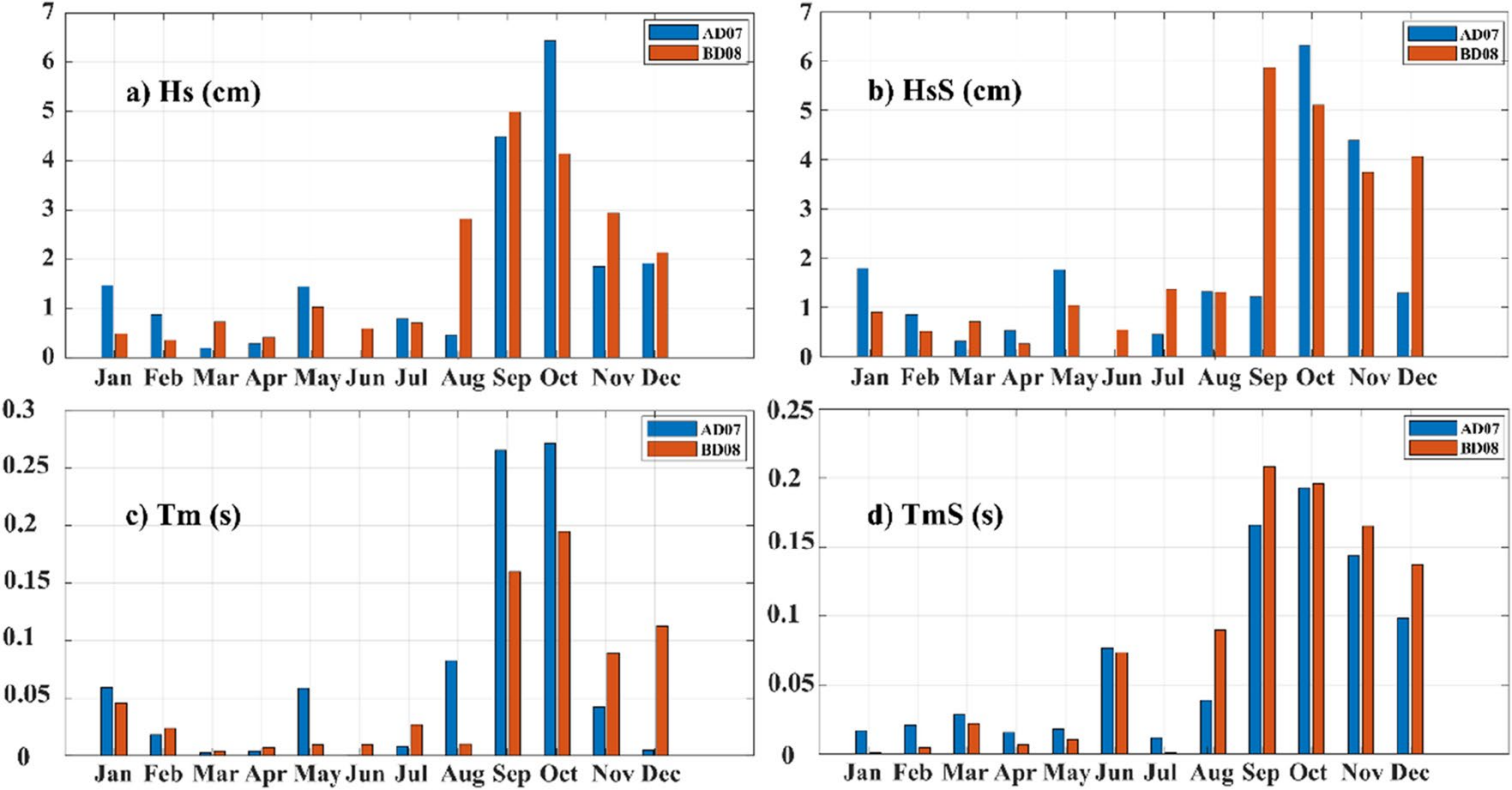
Monthly averaged difference of W3_*no_ice*_ and W3_*with_ice*_ for (**a**) significant wave height, Hs, (**b**) the swell wave height, HsS, (**c**) the mean wave period, Tm, and (**d**) the swell period, TmS at the AD07 (blue bars) and BD08 (red bars) mooring locations.

**Figure 3:**
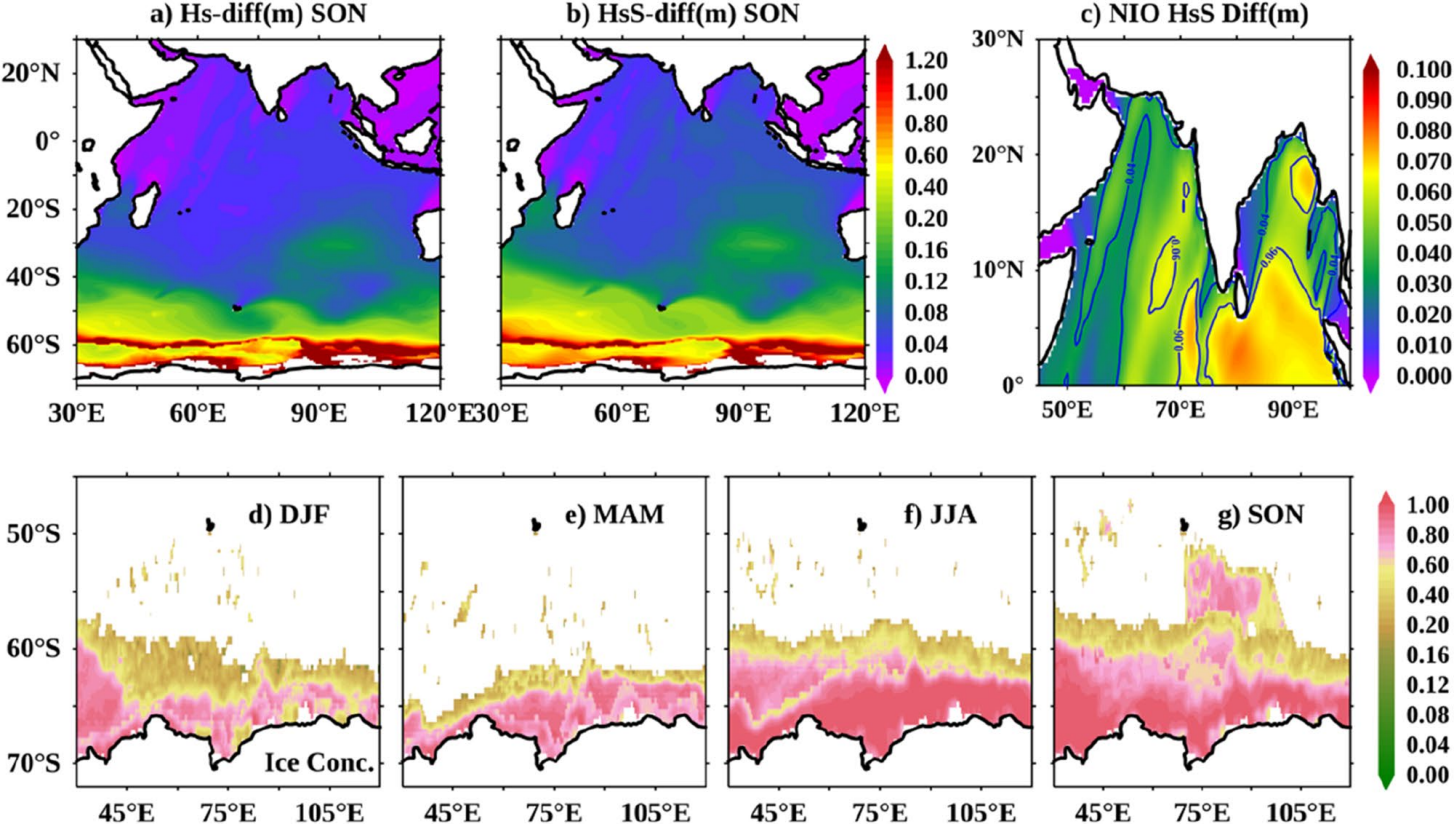
Maps showing SON averaged difference of W3_*no_ice*_ and W3_*with_ice*_ for (**a**) significant wave height, Hs, and (**b**) swell wave height, HsS. (**c**) is same as (**b**), but zoomed in for NIO basin. (**d–g**) Are the sea ice concentration in the Southern Ocean sector of Indian Ocean in different seasons. These seasonal averages are calculated for 2016–2021 period.

We conducted two experiments to quantify the impact of the SO sea ice concentration on the IO wave simulations. In the first experiment, we ran the WWIII model for the 2016–2021 period with the surface wind as the sole forcing field (W3_*no_ice*_), and in the second, WWIII was run with both winds and the SO sea ice concentration as the input fields (W3_*with_ice*_). The monthly averaged difference between W3_*no_ice*_ and W3_*with_ice*_ for a period of 6 years (2016–2021) in significant wave height (Hs), swell height (HsS), mean wave period (Tm), and swell period (TmS) at AD07 (blue bars) and BD08 (red bars) locations are shown in Fig. 2a–d. Figure 2 also shows that the difference in simulations in the total fields (Hs and Tm; Fig. 2a,c) are driven by the swell components (HsS and TmS; Fig. 2b,d), which is quite apparent. The maximum difference between W3_*no_ice*_ and W3_*with_ice*_ is observed in the SON period. The simulations without sea ice concentration generate low-frequency swells with more wave height in the NIO basins than simulations with sea ice concentration. The maximum monthly mean difference in Hs (HsS) between the two simulations (W3_*no_ice*_ − W3_*with_ice*_) in the NIO was observed in October, approximately 6.5 cm (6–7 cm), respectively. In the September–October period, a similar trend is noted in Tm and TmS in the NIO basins. The impact of sea ice concentration on the SO swell waves is maximum during the September–October–November (SON) season and minimum during March–April–May (MAM), coinciding positively with the spatial variation of sea ice concentration in the SO (Figs. 2 and 3). The spatial comparison of W3_*no_ice*_ – W3_*with_ice*_ in Hs and HsS for the SON period and the seasonal variation of sea ice concentration in the SO are shown in Fig. 3. The maximum difference of up to 2 m for Hs and HsS is seen in the extratropical south Indian ocean belt between 50–70° S (Fig. 3a,b). The HsS difference reaches ~ 8 cm in the northern BoB and retains a difference of ~ 6 cm through the NIO basin (except for the shadow regions north of Sri Lanka; Fig. 3c). This difference is on par with the monthly averaged difference seen at the buoy locations (Fig. 2).

**Figure 4:**
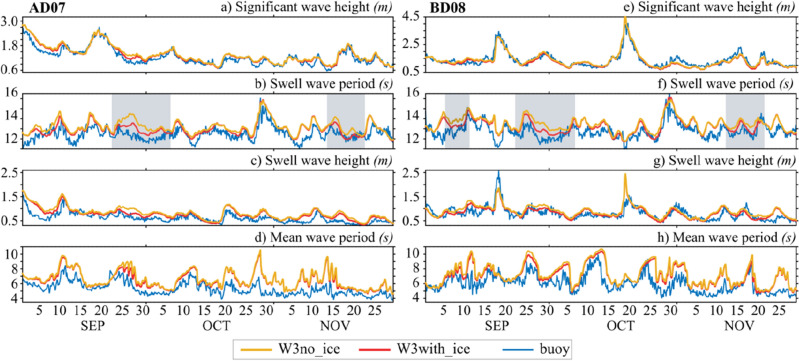
Time series comparison of W3_*no_ice*_ (orange) and W3_*with_ice*_ (red) with mooring data (blue) for (**a**,**e**) significant wave height, Hs, (**b**,**f**) swell wave period, Tm, (**c**,**g**) swell wave height, HsS and (**d**,**h**) mean wave period, Tm at AD07 (right panels) and BD08 (left panels) for 2017.

The difference between W3_no_ice_ and W3_with_ice_ simulations in the IO is not a clear indication of the accuracy of one over the other. We now compare the two simulations with the NIO mooring observations to see which simulation is closest to the observations. Figure 4 shows the time series comparison of wave parameters from W3_*with_ice*_ and W3_*no_ice*_ with NIO mooring observations during the SON period of 2017. There are times when there is a clear difference between the two simulations against in-situ data, and the W3_*with_ice*_ simulation consistently compares closest to observations. For example, during the 25 September-02 October 2017 period, at the AD07 location, the W3_*with_ice*_ simulation reproduced the swell height with ~ 50% less bias than the W3_*no_ice*_ simulation. Similar observations are noted in the Bay of Bengal at the BD08 location during the same period. Figure 4b,f also suggest similar results in swell period comparisons. It is noted that W3_*no_ice*_ simulations always produce high period swells (low frequency) propagating northwards. The total agreement of the model with the buoy cannot be seen in any of the wave parameters, which is expected due to the discrepancies involved in the forcing wind, ice concentration fields, the model parameterization scheme, etc.

**Figure 5:**
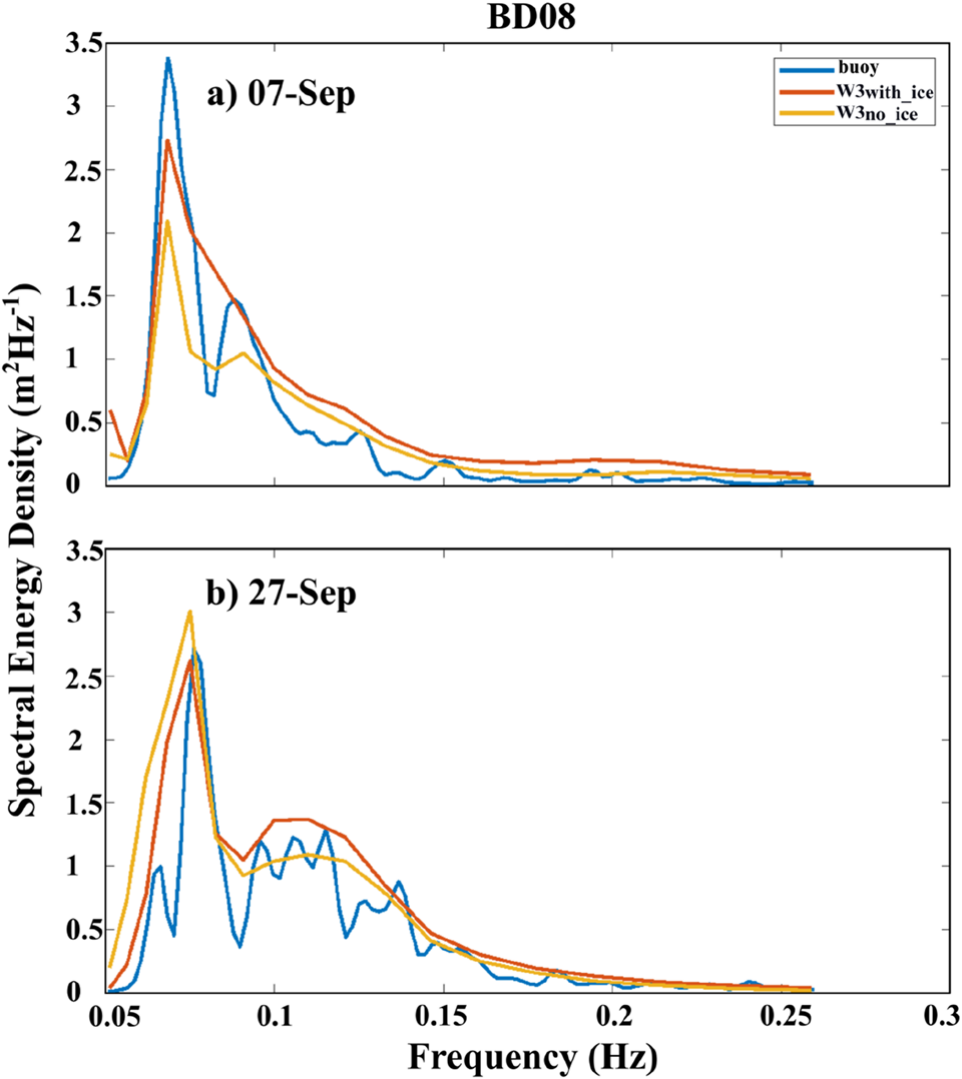
Spectral energy density comparison of W3_*no_ice*_ (orange) and W3_*with_ice*_ (red) with BD08 data (blue) at (**a**) 03 UTC on 07th September 2017 and (**b**) 09 UTCon 27th September 2017.

Table 1 provides the average statistics of W3_*with_ice*_ and W3_*no_ice*_ simulations against the six NIO mooring observations during the SON period of 6 years (2016–2021). The statistics shown are 95% significant (*p* = 0.05) using Student’s t-test. In all the NIO mooring locations we considered in this study, W3_*no_ice*_ HsS simulation biases are 8–10 cm more than W3_*with_ice*_ simulations compared to observations. We found that W3_*no_ice*_ is ~ 60% (37%) more biased than W3_*with_ice*_ simulations in HsS (TmS) averaged over the NIO mooring locations. A comparison of Tm and TmS suggests that W3_*no_ice*_ produces more low frequency (peak shift towards lower frequencies) swell waves compared to the swells in W3_*with_ice*_. The spectral energy data available for September 2017 at the BD08 location is used for the energy comparison between the two model simulations (Fig. 5a,b). The spectral peaks at low frequencies indicate swells, and W3_*no_ice*_ overestimates the energy density peak at ~ 0.07 Hz on 27th September 2017. Whereas W3_*with_ice*_ agrees well with observation, both models underestimate the low-frequency peak on 7th September, although W3_*with_ice*_ is better in comparison. At higher frequencies (> 0.15 Hz), W3_*no_ice*_ and W_3*with_ice*_ behave almost similarly. Overall, W3_*with_ice*_ is better at predicting low-frequency swells than the other simulation in the NIO basins.

**Table 1:** Validation statistics of significant wave height (Hs), swell wave height (HsS), wave period (Tm) and swell wave period (TmS), averaged over the six NIO mooring locations for the SON period of 2016–2021.

Year	Statistics	Variable							
		Hs		HsS		Tm		TmS	
		W3_*with_ice*_	W3_*no_ice*_	W3_*with_ice*_	W3_*no_ice*_	W3_*with_ice*_	W3_*no_ice*_	W3_*with_ice*_	W3_*no_ice*_
2016–2021 (n = 8774)	*Bias(cm)*	− 1.00	6.00	4.00	13.00	0.96	1.11	0.49	0.78
	*corr*	0.94	0.94	0.83	0.83	0.72	0.70	0.68	0.65
	*rmse*	0.21	0.22	0.20	0.24	1.41	1.56	0.80	1.03
	*SI*	0.14	0.15	0.25	0.30	0.24	0.26	0.06	0.08

The statistics are valid at 95% confidence level (*p* = 0.05).

**Figure 6:**
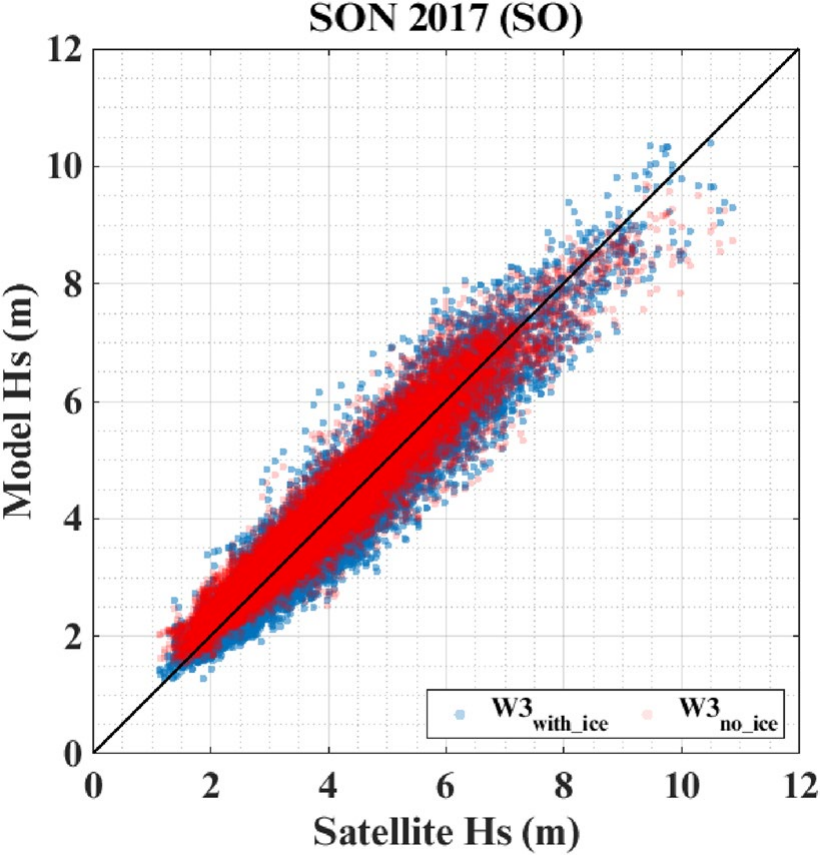
Satellite Hs comparison with W3_*with_ice*_ and W3_*no_ice*_ simulations for the ice-free regions of SO (55–40° S, 40–110° E) for SON of 2017.

In-situ observations are unavailable from the SO for wave field comparison between the two simulations. One possible option to ascertain the quality of wave simulations in the SO is to compare the Hs from the satellite with the model simulations. Figure 6 shows the satellite Hs comparison with W3_*with_ice*_ and W3_*no_ice*_ simulations for the ice-free regions of the SO (55–40° S, 40–110° E) for SON of 2017. Both W3_*with_ice*_ and W3_*no_ice*_ simulations are correlated well with satellite Hs, but W3_*with_ice*_ Hs exhibit more scatter than W3_*no_ice*_ Hs. One noticeable feature is the overestimation (~ 20 cm) of mean Hs by W3_*no_ice*_ in the lower ranges, which are mainly the developing seas. Hence, similar to the wave field comparisons in the NIO with mooring data, satellite Hs comparison in the ice-free regions of the SO suggests that W3_*with_ice*_ simulates Hs better than W3_*no_ice*_.

## Implications of SO sea ice concentration on wave simulations

In the previous section, we presented the numerical difference in the wave fields between W3_*with_ice*_ and W3_*no_ice*_ simulations and found that W3_*with_ice*_ simulates accurate wave parameters. This section shows the local and remote implications of the SO sea ice concentration inclusion as an input field in the wave simulations. Figure 3d–g show the average sea ice concentration for different seasons used to force the model to run the W3_*with_ice*_ simulation. Around 1,00,00 km^2^ area is covered with sea ice concentration in the southern sector of IO during SON, which acts as a large fetch area for wave generation in W3_*no_ice*_ simulation. The area around 30–50° E and 70–50° S is considered a strong swell generating area^14^ with a fetch size of ~ 44,000 km^2^. In W3_*no_ice*_, the entire 44,000 km^2^ is available as fetch for swell generation, while in W3_*with_ice*_, ~ 46% of the area is ice-covered during SON (see Fig. 3g). Considering a fetch area of 44,000 km^2^, a friction velocity of 0.46 m/s at an average wind speed of 12 m/s, the average Hs calculated will be 1.27 m in W3_*no_ice*_ using the following equation:

$$\frac{{gH_{s} }}{{u_{f}^{2} }} = 4.13*10^{ - 2} \left[ {\frac{gX}{{u_{f}^{2} }}} \right]^{0.5} ;$$

where *X* is the fetch area, *g* is the acceleration due to gravity, *u*_*f*_ is the friction velocity, and *Hs* is the significant wave height. Repeating the wave height calculation for W3_*with_ice*_ simulation with 46% less fetch area produces a wave height of 0.86 m. A fetch reduction of ~ 46% on introducing sea ice concentration causes a 32% reduction in wave height in W3_*with_ice*_. These calculations agree with the satellite Hs comparisons in the previous section, where W3_*no_ice*_ simulated higher Hs compared to W3_*with_ice*_ simulations.

Following the classical wave theory, the group velocity of deep water waves is Cg = 0.78 T m/s, where T is the wave period. On a general note, taking the average difference in swell wave period between W3_*no_ice*_ and W3_*with_ice*_ as 0.15 s (Fig. 2b), the ratio of Cg between W3_*no_ice*_ and W3_*with_ice*_ suggests that W3_*no_ice*_ swells travel faster by ~ 11.5% than W3_*with_ice*_ swells. This speed difference has significant implications. Most SO swell waves travel ~ 9000 km to reach NIO coastal regions and have a typical phase speed of ~ 20 m/s. Thus, the SO swells take ~ 5.2 days to reach the NIO coastal regions in W3_*with_ice*_ simulations. An 11.5% increase in the phase speed in W3_*no_ice*_ compared to W3_*with_ice*_ suggests that W3_*no_ice*_ swells take only 4.67 days to reach NIO coastal regions. The difference of ~ 12 h in predicting the SO swell impact on NIO coastal regions can sometimes be disastrous in operational wave forecasting.

**Figure 7:**
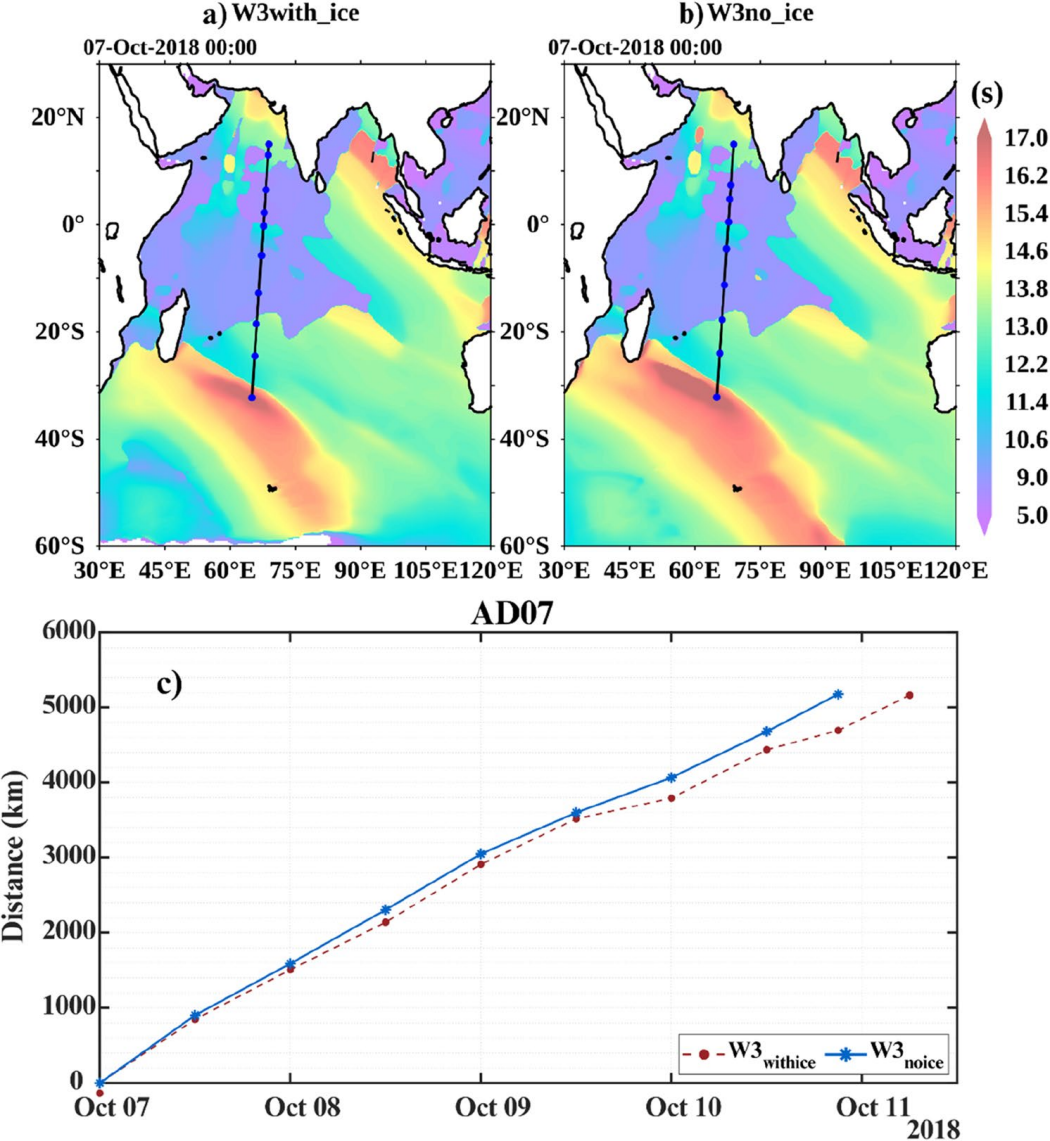
Distance line of swell from SO to AD07 mooring location from 07-Oct-2018 00 h with blue dots representing 12-h marks in (**a**) W3_*with_ice*_ and (**b**) W3_*no_ice*_ simulations and (**c**) shows the distance versus time of the swell system in W3_*no_ice*_ and W3_*with_ice*_ over the next few days until arrival at AD07 location.

Here we consider a typical case of a swell system that was generated in the SO on 7th October 2017 and travelled northwards into the NIO. Figure 7a,b show the approximate origin of the swell system in the SO in W3_*with_ice*_ and W3_*no_ice*_ simulations. The straight line indicates the typical distance the swell wave travelled, ~ 6400 km, to reach the AD07 mooring location in the Arabian Sea, and the dots in the line indicate the distance the swell system reached along the straight line every 12 h. Figure 7c shows the distance vs. time of the swell system in W3_*no_ice*_ and W3_*with_ice*_ over the next few days until it reached the AD07 location. The W3_*no_ice*_ swell system travelled faster than W3_*with_ice*_ and reached the mooring location on 10th October 2018 at  21 h, while W3_*with_ice*_ swells were slower and reached AD07 on 11th October 2018 at 9 h. Thus there is a net difference of 12 h between the swell system of W3_*with_ice*_ and W3_*no_ice*_ to travel from its source region in the SO to reach the AD07 location. These observations are in agreement with our theoretical calculations provided above. In the previous section, a comparison of the two simulations with observations suggests that W3_*no_ice*_ produces low-frequency swells that travel faster. Here, we have shown the typical case of such a system with an arrival time difference of ~ 12 h at the AD07 location.

Another noticeable feature of Fig. 7a,b is the arc length of the swell system generated from the southwestern sector of the IO. The swell generating area of the IO is concentrated mainly on the southwest side of the SO^13^, where the sea ice concentration undergoes a prominent seasonal cycle (Fig. 3d–g.). Due to the larger fetch area, W3_*no_ice*_ produces a much bigger swell system that sweeps over a wider geographical extent of the eastern portion of IO, especially the southwestern coastal regions of Australia. The arc length of W3_*with_ice*_ is shorter than W3_*no_ice*_ and has limited impact along Australian coasts. Thus W3_*no_ice*_ could potentially produce false swell alerts along such regions.

In short, our analysis in this section shows that the ice cover in the SO will reduce the fetch, affecting wave growth. We also see a reduction in the swell wave period if sea ice concentration is included in the SO. The decrease in the wave period will change the arrival time of such swells on NIO coasts, which is critical from a forecasting point of view^18^. Failure to accurately predict the timing of such swell surges, which usually do not have any local weather changes along NIO coasts, can pose a significant threat to the lives and livelihoods of the coastal population.

## Summary and discussion

This article explores the effect of the Southern Ocean (SO) sea ice concentration on the Indian Ocean (IO) wave conditions using WAVEWATCHIII (WWIII) numerical wave model simulations. The impact of the SO sea ice concentration on IO swells is not explicitly addressed in scientific literature. However, past studies on IO wave climate proved the effect of SO swells on IO coastal areas. In the present study, we did two WWIII model simulations, one with winds and the SO sea ice concentration as forcing fields (*W3*_*with_ice*_) and the other with only winds as forcing (*W3*_*no_ice*_), to explore the effect of the SO sea ice concentration on wave simulations. Our analysis suggests a non-negligible difference in HsS and TmS in the SO (Fig. 2b,d). Validations with NIO mooring data suggest that wave simulations with the SO sea ice concentration accurately reproduce the observed wave characteristics, both Hs and Tm. The impact of sea ice concentration on the SO swell waves is maximum during the September–October–November (SON) season and minimum during March-April-May (MAM), coinciding positively with the spatial variation of sea ice extent in the SO.

The impact of sea ice concentration is reflected as a few centimeters (~ 6–8 cm; Fig. 3c) change in swells height in the NIO wave simulations. Generally, W3_*no_ice*_ simulates low-frequency swells that travel fast towards NIO. Our analysis suggests that WWIII simulations with sea ice concentration accurately forecast the timing of high swell events in the NIO coastal regions. We tracked a SO swell system formed on 7th October 2018, in the two simulations and analyzed the arrival time at the AD07 mooring location in the Arabian Sea, which is ~ 6400 km from the swell wave formation. We found that swells from W3_*wtih_ice*_ reached first at AD07 in 105 h, while swells from W3_*no_ice*_ simulation took 93 h to arrive. This difference of ~ 12 h in the arrival time is also proved theoretically in “Implications of SO sea ice concentration on wave simulations section. Due to the larger fetch area, W3_*no_ice*_ produces a much bigger swell system that sweeps over a wider geographical extent of the eastern portion of IO, especially the southwestern coastal regions of Australia. Hence it is presumed that W3_*no_ice*_ could potentially produce false swell alerts along such southeastern Australian coasts. Overall, our analysis suggests that W3_*wtih_ice*_ simulates well the NIO swells. Accurate forecasts of swell surges and catastrophic events like Kallakkadal will help the disaster management authorities to coordinate well with the local civil administration to handle the response activities in the coastal regions. Moreover, it asserts faith in the coastal population to take such forecasts seriously and corporate with the response coordination of the disaster management authorities.

A potential caveat of our analysis is the lack of model validation in the SO due to the non-availability of in situ observations in such remote regions. Comparing simulated wave fields with NIO mooring observations suggests a good correlation and minimum bias in wave fields. This comparison indirectly indicates that the swell simulation from the SO is probably correct, an inference positively supported by the satellite Hs comparison presented in this paper. Further, our analysis included the year 2017, the record low in Southern hemisphere sea ice extent. A long-term analysis may be required beyond the 6 years (2016–2021) we used here.

WWIII employs several wave-ice parameterization schemes (like IC0, IC1, IC2, IC3, IC4, and IC5) to account for the effect of sea ice concentration in wave simulations^23^. We did not focus on validating different wave-ice parameterization schemes here but selected the simple IC0 scheme, which provides energy flux blocking depending on the local ice concentration and does not consider the thickness of the ice. Studies suggest that the simulation of wave fields under ice-covered conditions relies on the accuracy of sea ice concentration data used as a model forcing and the accuracy of the wave-ice parameterization^23^. Future studies may require validating the sea ice concentration data and the wave-ice parameterizations.

The sea ice extent in different seasons in the Indian sector of the SO varies largely, from a minimum of 74,208.5 km^2^ in DJF to a maximum of 1,08,308.75 km^2^ in SON, and a wave model without this large sea ice extent will grossly overestimate the wave growth. Hence our arguments in this article will be valid irrespective of the above caveats, and any change in the ice extent in the SO will substantially impact NIO through the SO swells. Thus our study recognizes sea ice concentration in the SO as a critical factor in modifying wave characteristics in the NIO and underscores the need to include it in wave modeling for forecasting and climate simulations.

## Data Availability

The daily sea-ice concentration data from The https://n5eil01u.ecs.nsidc.org/SMAP_ANC/SMAP_L1_L3_ANC_NOAA.001/.E The bathimetry data used is from NOAA National Geophysical Data Center. 2009: ETOPO1 1 Arc-Minute Global Relief Model. NOAA National Centers for Environmental Information. Accessed (https://www.ngdc.noaa.gov/mgg/global/relief/ETOPO1/data/ ). The mooring observations used extensively in this article can be accessed upon request from INCOIS (https://incois.gov.in/portal/datainfo/drform.jsp).

## References

[1] Stopa JE, Sutherland P, Ardhuin F (2018). Strong and highly variable push of ocean waves on Southern Ocean sea ice. Proc. Natl. Acad. Sci..

[2] Marshall J, Speer K (2012). Closure of the meridional overturning circulation through Southern Ocean upwelling. Nat. Geosci..

[3] Sallée JB (2018). Southern Ocean warming. Oceanography.

[4] The IMBIE team (2018). Mass balance of the Antarctic Ice Sheet from 1992 to 2017. Nature.

[5] Squire VA (2018). Fresh look at how ocean waves and sea ice interact. Philos. Trans. R. Soc. A.

[6] Asplin MG, Galley R, Barber DG, Prinsenburg S (2012). Fracture of summer perennial sea ice by ocean swell as a result of Arctic storms. J. Geophys. Res..

[7] Collins CO, Rogers WE, Marchenko A, Babanin AV (2015). In situ measurements of an energetic wave event in the Arctic marginal ice zone. Geophys. Res. Lett..

[8] Ardhuin F, Otero M, Merrifield S, Grouazel A, Terrill E (2020). Ice breakup controls dissipation of wind waves across southern ocean sea ice. Geophys. Res. Lett..

[9] Masson D, LeBlond P (1989). Spectral evolution of wind-generated surface gravity waves in a dispersed ice field. J. Fluid Mech..

[10] Shen HH, Hibler WD, Leppäranta M (1986). On applying granular flow theory to a deforming broken ice field. Acta Mech..

[11] Herman A (2013). Numerical modeling of force and contact networks in fragmented sea ice. Ann. Glaciol..

[12] Barbariol F, Benetazzo A, Bertotti L, Cavaleri L, Durrant T, McComb P, Sclavo M (2019). Large waves and drifting buoys in the Southern Ocean. Ocean Eng..

[13] Vichi M, Eayrs C, Alberello A, Bekker A (2019). Effects of an explosive polar cyclone crossing the Antarctic marginal ice zone. Geophys. Res. Lett..

[14] Zheng CW, Li CY, Pan J (2018). Propagation route and speed of swell in the Indian Ocean. J. Geophys. Res. Oceans.

[15] Zheng CW, Shao LT, Shi WL, Su Q, Lin G, Li XQ, Chen XB (2014). An assessment of global ocean wave energy resources over the last 45 a. Acta Oceanol. Sin..

[16] Sabique L, Annapurnaiah K, Balakrishnan Nair TM, Srinivas K (2012). Contribution of Southern Indian Ocean swells on the wave heights in the Northern Indian Ocean—A modeling study. Ocean Eng..

[17] Swapna P, Ravichandran M, Nidheesh G, Jyoti J, Sandeep N, Deepa JS (2020). Assessment of Climate Change over the Indian Region A Report of the Ministry of Earth Sciences (MoES).

[18] Remya PG, Vishnu S, Praveen Kumar B, Nair TMB, Rohith B (2016). Teleconnection between the North Indian Ocean high swell events and meteorological conditions over the Southern Indian Ocean. J. Geophys. Res. Oceans.

[19] Chawla A (2007). A multigrid wave forecasting model: A new paradigm in operational wave forecasting. Weather Forecast..

[20] Ardhuin F (2010). Semiempirical dissipation source functions for ocean waves. Part I: Definition, calibration, and validation. J. Phys. Oceanogr..

[21] Tolman HL (2003). Treatment of unresolved islands and ice in wind wave models. Ocean Model.

[22] Premkumar K, Ravichandran M, Kalsi SR, Sengupta D, Gadgil S (2000). First results from a new observational system over the Indian seas. Curr. Sci..

[23] Iwasaki S, Otsuka J (2021). Evaluation of wave-ice parameterization models in WAVEWATCH III R© along the coastal area of the sea of Okhotsk during winter. Front. Mar. Sci..

